# Mid-level health providers for primary healthcare: a rapid evidence synthesis

**DOI:** 10.12688/f1000research.24279.1

**Published:** 2020-06-16

**Authors:** Sandeep Moola, Soumyadeep Bhaumik, Devaki Nambiar

**Affiliations:** 1 George Institute for Global Health, Vishakhapatnam, India; 2 The George Institute for Global Health, Faculty of Medicine, University of New South Wales, Sydney, New South Wales, Australia

**Keywords:** Rapid review, rapid evidence synthesis, systematic reviews, mid-level health providers, MLHPs, low- and middle-income countries

## Abstract

**Background: **Health care services, in many countries, are increasingly being provided by cadres not trained as physicians, but capable of performing several diagnostic and clinical functions. These substitute health workers are referred to as mid-level health providers (MLHPs). The health and wellness centres under India’s Comprehensive Primary Health Care programme have teams led by MLHPs who can aid doctors. The objective of this study was to rapidly synthesise evidence on the effectiveness of MLHPs for primary health care.

**Methods:** The review team undertook a rapid overview of systematic reviews that compared MLHPs with doctors and different types of MLHPs involved in the delivery of health care were included, with a perspective on low- and middle-income countries, including India.

**Results: **Seven systematic reviews were included in the final report. Mortality outcomes in relation to pregnancy and childbirth care services showed no significant differences in care provided by MLHPs when compared with doctors. Pregnancy care provided by midwives was found to slightly improve quality of care when compared to care delivered by doctors. The risk of failure or incomplete abortion for surgical abortion procedures provided by MLHPs was twice when compared to the procedures provided by doctors. Moderate to high certainty evidence showed that initiation and maintenance of antiretroviral therapy for HIV-infected patients by a nurse or clinical officer slightly reduced mortality. High certainty evidence showed that chronic disease management by non-medical prescribers reduced some important physiological measures when compared to medical prescribing by doctors.

**Conclusions:** To date, this is the first rapid overview of evidence on MLHPs. Evidence suggests that MLHPs might be suitable to deliver quality care in certain areas of health and they may be relevant and feasible in countries like India. However, the roles and subsequent training and regulation of MLHPs might be different for different care domains.

## Introduction

There is a growing momentum worldwide to improve access to healthcare and provide efficient and cost-effective primary health care (PHC) ^1^. Mid-level health providers (MLHPs) are currently being used in high- and low-income countries to assist doctors and specialists and/or to render services independently, particularly in resource-poor settings to make up for the scarcity of health professionals. A cornerstone of current health systems reform efforts in India is the flagship *Ayushman Bharat* (AB) program. Largely the program has an insurance component (Pradhan Mantri Jan Arogya Yojana, PMJAY) and development of Health and Wellness Centres (HWCs) as strategies to advance on the path to universal health coverage ^2^.

Ayushman Bharat’s HWC sub-strategy, the comprehensive primary health care (CPHC), conceives of MLHPs as a key focal point for service organisation and delivery, performing a range of screening, diagnostic and clinical functions and improve health systems at the frontline. The program conceptualises 12 different packages for the CPHC reforms ^3^. One key pillar of rolling out the AB-HWC component is the inclusion of new health cadre trained and accredited for a set of skills/competencies related to PHC and public health. Further, one of the aims of this programme is the transformation of existing sub-health centres and PHCs to HWCs, with teams led by MLHPs.

The National Health Systems Resource Centre (NHSRC), the technical support agency of the National Health Mission, is responsible for developing the curriculum for MLHPs. We received a request from the NHSRC for a rapid review of evidence on the effectiveness of MLHPs in the PHC context of low- and middle-income countries (LMICs) to understand the role MLHPs can play in different packages. We host a rapid evidence synthesis (RES) platform, which provides gratis RES to public agencies. RES or rapid review is an emerging form of evidence synthesis that is increasingly being promoted by the WHO and employed by governments to inform decision making ^4^. We thus synthesised evidence related to the effectiveness of MLHPs in the PHC context of LMICs.

## Methods

### Approach for RES

We conducted a rapid overview of systematic reviews (SRs) of evidence on the effectiveness of MLHPs within a span of about eight weeks and in all domains corresponding to the CPHC package in *Ayushman Bharat*. The 12 CPHC packages are: pregnancy and childbirth; neonatal and infant health services; childhood and adolescent health services; family planning, contraceptive services and other reproductive care services; communicable diseases (prevention and management); non-communicable diseases; elderly and palliative care; oral health care; ophthalmic and ear, nose and throat (ENT) care; mental health and emergency medical services ^3^.

The World Health Organization (WHO), defined MLHP as “a health provider who is trained, authorised and regulated to work autonomously, receives pre-service training at a higher education institution for at least 2–3 years and whose scope of practice includes (but is not restricted to) being able to diagnose, manage and treat illness, disease, and impairments (including performing surgery, where appropriately trained), prescribe medicines, as well as engage in preventive and promotive care” ^1, p.8^. However, MLHPs in various countries have been variously referred to as substitute health workers, auxiliaries, non-physician clinicians, and include cadres such as clinical officers, medical assistants, physician assistants, nurse practitioners, and surgical technicians. Institutions and researchers worldwide use alternate or less well-specified definitions, and therefore MLHP as defined in the SRs was considered for this review. Therefore, we used broad criteria for the rapid overview wherein we accepted the definition of MLHPs as defined by the SR authors. The overview of SRs is an appropriate study design for our research because we intended to summarise the evidence for multiple conditions in different disease/condition domains for the same type of intervention and on similar health systems, clinical and public health outcomes.

### Inclusion criteria

***Participants.*** The RES considered SRs assessing outcomes for participants receiving care from MLHPs in LMICs, including India.

***Intervention and comparators.*** SRs that compared service delivery provided by MLHPs with doctors or other types of MLHPs were included. The MLHPs included were midwives, nurses, auxiliary nurses, nurse assistants, non‐physician clinicians, and surgical technicians.

***Outcomes.*** The following outcomes were considered for inclusion based on the initial discussions with the requester: healthcare and clinical outcomes (mortality, morbidity, outcomes associated with care delivery, and physiological measures); access to care; and quality of care (including patient or client satisfaction with care).

***Study design.*** SRs including studies of any quantitative study design, irrespective of whether they have or have not conducted meta-analyses and irrespective of whether they have or have not used the Grading of Recommendations Assessment, Development and Evaluation (GRADE) framework to assess the certainty of evidence were included. Qualitative SRs were not considered.

***Context.*** SRs with a focus on and/or including studies from LMICs and India were included.

### Search strategy

Given time constraints, the search was limited to published and indexed articles, and those published in the English language. The following databases were searched (from database inception up until March 2019): Cochrane Database of Systematic Reviews; Medline (PubMed); EMBASE; Health Systems Evidence; and CINAHL. An additional search was conducted from April 2019 to April 2020 to update the review findings for recency and relevancy. Search strategies (for both the periods) are provided separately for each database (see *Extended data*) ^5^.

### Data collection and analysis

The lead reviewer (SM) independently screened the titles and abstracts of studies for inclusion, following which full-text examination of eligible studies was conducted for potential inclusion. A second reviewer (SB) randomly verified the results of the study selection process during both the screening stages. For each domain of interest, where multiple SRs were available, only one SR was included based on its comprehensiveness, recency, and quality. Each SR was independently assessed for methodological quality by using established standardised criteria (A MeaSurement Tool to Assess systematic Reviews (AMSTAR) 2 checklist) ^6^. Data from included reviews was extracted using a pre-defined template, which included variables such as review type, review question, countries/settings, participants characteristics, interventions, outcome measures and review conclusions. The lead author (SM) independently extracted all relevant outcome data, with random verification of 20% of the included studies by another author (SB).

### Summary of findings

The GRADE approach was used to assess the certainty of the evidence using a transparent framework for developing and presenting the summary of findings tables ^7– 10^. The GRADE of evidence was synthesised with respect to a PHC setting and in an LMIC context to make the product locally relevant ^9, 10^.

### Stakeholder engagement

As part of the RES process, the RES team and NHSRC jointly convened a policy dialogue to engage and consult with relevant stakeholders to present an interim draft of the MLHP policy brief. The stakeholders included policy makers (key stakeholders from government agencies and collaborators), health system managers, and researchers from more than eight states in India.

## Results

### Search results and study selection

**Figure 1.**
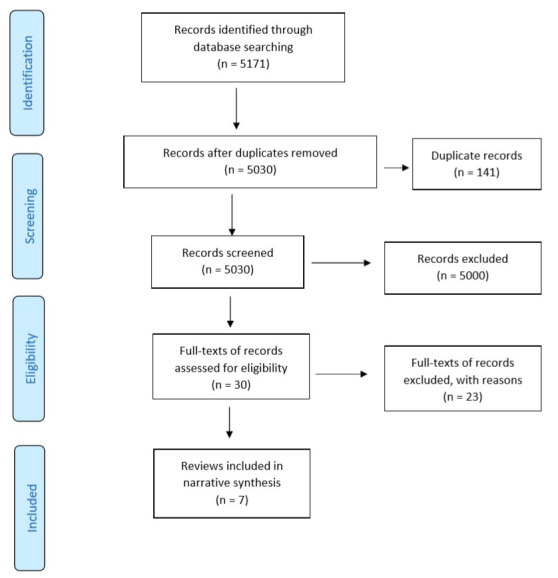
PRISMA study flow diagram. Search conducted from database/s inception up until March 2019.

**Figure 2.**
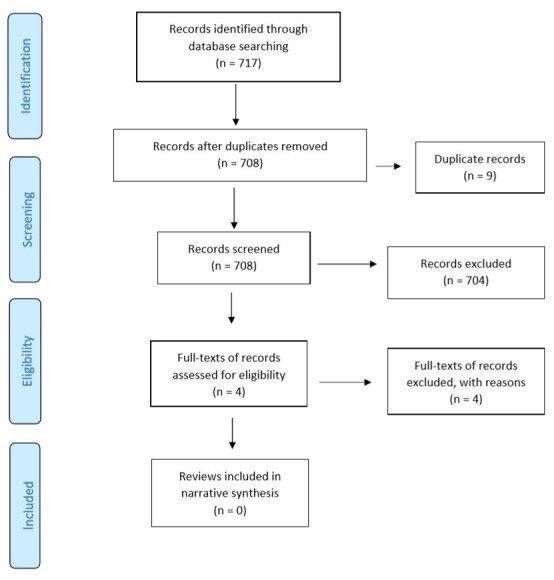
PRISMA study flow diagram (updated search). Updated search from April 2019 to April 2020.

The search for evidence identified 5171 studies ( Figure 1 – Preferred Reporting Items for Systematic Reviews and Meta-Analyses (PRISMA) flow diagram). Following the study screening process, full-text articles were retrieved for 30 potentially relevant studies. In cases where there were multiple SRs for the same domain, the SR that was the most recent and provided comprehensive information (as per authors’ (SM, SB) consensus) was selected and included. Following full-text examination, 23 out of 30 SRs were excluded. An additional 717 records were identified in an updated search. However, following the study selection process, none of the reviews were found to be relevant to the topic of interest ( Figure 2 – PRISMA flow diagram (updated search)). Overall, seven SRs were included in the RES.

### Characteristics of included SRs

The majority of the studies included in the SRs were randomised controlled trials (RCTs), with some quasi-experimental study designs and observational studies. Key characteristics of the included SRs are provided in the *Extended data* file ^5^. All SRs, except one ^11^ included studies that were mostly conducted in LMICs. The studies related to HIV/AIDS were mostly conducted in sub-Saharan African countries. Most studies compared care provided by midwives or auxiliary nurse midwives or nurses with that provided by doctors working in a team along with midwives or nurses.

### Methodological quality of included SRs

The AMSTAR-2 checklist ^6^ was used to assess the methodological quality of SRs included in the report. The checklist is a 16-item questionnaire. The critical appraisal results of the included SRs are provided in the *Extended data* file ^5^. Six out of seven SRs were of moderate to high methodological quality and well reported. Almost all the SRs did not refer to *a priori* protocol and publication bias was not assessed. One SR by Chaudhary *et al.* was of poor quality, as assessed by the checklist ^12^.

### Summary of findings tables for each domain of interest

***Key findings.*** The key findings from the included SRs have been categorised based on the various healthcare domains of interest in the CPHC package ^3^. The quality of evidence for the main outcomes is summarised using the GRADE approach and ‘Summary of Findings’ tables ^7– 10^. The Summary of Findings tables aid in recording results, outcomes, and outcome risks in a structured synthesis format.

***MLHPs for care in pregnancy and childbirth.*** An SR compared the effectiveness of care provided by MLHPs, particularly midwives and auxiliary nurse midwives with doctors providing care in a team with midwives ^11^. The review included patients receiving pregnancy and childbirth services including antenatal care. The majority of the studies were conducted in tertiary care settings and developed countries. Most of the evidence was assessed as low certainty. It was found that the use of intrapartum analgesia and episiotomies were less likely with care provided by midwives when compared with that provided by doctors working along with midwives. Also, no significant difference in rates for performing caesarean section, postpartum haemorrhage, and preterm births were reported.

No significant difference in the likelihood of an incomplete abortion was reported between groups of patients treated by auxiliary nurse midwives compared to those cared for by doctors. However, the likelihood of a complication during or an adverse event after manual vacuum aspiration was significantly greater with care provided by auxiliary nurse midwives. There was very low certainty evidence to suggest that pregnancy care provided by clinical officers reduced the likelihood of early neonatal death or postoperative maternal health outcomes, such as fever and wound infections. Table 1 provides a summary of findings and certainty of evidence related to pregnancy and childbirth care provided by midwives, auxiliary nurse midwives and clinical officers with that provided by doctors in a team with midwives.

**Table 1.** Summary of findings for care provided by MLHPs for pregnancy and childbirth.

	Outcomes	Relative effect (95% CI)	No of participants	Certainty of the evidence (GRADE)	Plain language summary
Midwives alone versus doctors along with midwives					
**Randomised Controlled Trials (RCTs)**	Rate of performing caesarean sections	RR 0.94 (0.81 to 1.06)	12144 (8 RCTs)	⨁⨁◯◯ Low ^1^	Pregnancy care provided by midwives may slightly reduce the rate of performing caesarean sections (low certainty evidence)
	Postpartum haemorrhage	RR 0.53 (0.25 to 1.14)	8604 (6 RCTs)	⨁⨁◯◯ Low ^1, 2^	Pregnancy care provided by midwives may reduce postpartum haemorrhage (low certainty evidence)
	Preterm births	RR 0.87 (0.73 to 1.04)	9210 (5 RCTs)	⨁⨁◯◯ Low ^1^	Pregnancy care provided by midwives may slightly reduce preterm births (low certainty evidence)
	Use of intrapartum regional analgesia	RR 0.87 (0.81 to 0.93)	9415 (8 RCTs)	⨁⨁◯◯ Low ^1^	Pregnancy care provided by midwives may slightly reduce the use of intrapartum regional analgesia (low certainty evidence)
	Episiotomies	RR 0.85 (0.78 to 0.92)	13205 (8 RCTs)	⨁⨁◯◯ Low ^1^	Pregnancy care provided by midwives alone may slightly reduce in episiotomies (low certainty evidence)
	Quality of care (QoC)	RR 1.23 (1.10 to 1.37)	826 (1 RCT)	⨁⨁◯◯ Low ^1, 3^	Pregnancy care provided by midwives may slightly improve quality of care (low certainty evidence)
	Mortality and Access to care	-	-	-	No studies were found that examined these outcomes
Auxiliary nurse midwives versus doctors					
**RCTs**	Incomplete abortion	RR 0.93 (0.45 to 1.90)	1032 (1 RCT)	⨁⨁◯◯ Low ^1, 3^	Pregnancy care provided by auxiliary nurse midwives may make little or no difference in the likelihood of an incomplete abortion (low certainty evidence)
	Complications during conduct of manual vacuum aspiration	RR 3.07 (0.16 to 59.1)	2789 (1 RCT)	⨁⨁◯◯ Low ^1, 3^	Pregnancy care provided by auxiliary nurse midwives may make little or no difference in complications during manual vacuum aspiration. However, the wide 95% confidence interval includes the possibility of both increased and reduced complications (low certainty evidence)
	Post-operative adverse event	RR 1.36 (0.54 to 3.40)	2761 (1 RCT)	⨁⨁◯◯ Low ^1, 3^	Pregnancy care provided by auxiliary nurse midwives may increase post-operative adverse events; however, the 95% confidence interval includes the possibility of both increased and reduced postoperative adverse events (low certainty evidence)
	Clinical officers versus doctors					
**Observational studies**	Likelihood of early neonatal death	RR 1.40 (0.51 to 3.87)	(1 observational study)	⨁◯◯◯ Very low ^4^	It is uncertain whether pregnancy care provided by clinical officers reduces the likelihood of early neonatal death as the certainty of the evidence has been assessed to be very low
	Postoperative maternal health outcomes, such as fever, wound infection, the need for re- operation and maternal death, after emergency obstetric procedures	RR 0.99 (0.95 to 1.03)	(1 observational study)	⨁◯◯◯ Very low ^4^	It is uncertain whether pregnancy care provided by clinical officers reduces the effect on postoperative maternal health outcomes as the certainty of the evidence was assessed to be very low

^1^Downgraded one level due to serious risk of bias and another two levels due to indirectness (almost all the studies were conducted in tertiary care centres and high-income countries).

^2^Downgraded one level due to serious inconsistency (considerable heterogeneity was found).

^3^Downgraded one level due to imprecision (single study with a small sample size yielding wide confidence intervals spanning line of no effect).

^4^Quality of evidence was downgraded from Low (observational study design) to Very low due to a very serious risk of bias.

CI, confidence interval; GRADE, Grading of Recommendations, Assessment, Development and Evaluations; RR, risk ratio; RCT, randomised controlled trial; QoC, quality of care; MLHPs, mid-level health providers.

***MLHPs for neonatal and infant health care services.*** The effectiveness of midwives/nurses delivering care for neonatal and infant healthcare services was compared with that provided by doctors or obstetricians in a team with midwives in a SR ^11^. The population included patients receiving neonatal and infant health services. The majority of the studies were conducted in tertiary care settings and developed countries. The certainty of the evidence was assessed as low quality. The review results showed that there was no significant difference between the groups in foetal or neonatal death rates. None of the studies included in the review reported on clinical outcomes, and outcomes related to quality of care and access to care. Table 2 presents the review findings in plain language format and the certainty of the evidence for the relevant outcome.

**Table 2.** Summary of findings for care provided by MLHPs for neonatal and infant health care services.

	Outcomes	Relative effect (95% CI)	No of participants	Certainty of the evidence (GRADE)	Plain language summary
Midwives versus obstetrician or doctor in team with midwives					
**RCTs**	Foetal or neonatal death	RR 0.94 (0.56 to 1.58)	11562 (6 RCTs)	⨁⨁◯◯ Low ^1^	Care provided by midwives alone may result in little to no difference in foetal or neonatal deaths (low certainty evidence)
	Clinical outcomes; quality of care & access to care	-	-	-	No studies were found that examined these outcomes

^1^Downgraded one level due to serious risk of bias and two levels due to indirectness (almost all the studies were conducted in tertiary care centres).

CI, confidence interval; GRADE, Grading of Recommendations, Assessment, Development and Evaluations; RR, risk ratio; RCT, randomised controlled trial; MLHPs, mid-level health providers.

***MLHPs for family planning, contraceptive and other reproductive health care services.*** Another SR by Barnard *et al.* evaluated the safety and effectiveness of surgical and medical abortion procedures administered by MLHPs compared to doctors ^13^. The review included various MLHPs who included nurses, midwives, doctor assistants, and physician assistants delivering care for patients requesting abortion procedures, either surgical or medical. The majority of the studies were conducted in PHC settings and LMICs. Much of the evidence was of low or very low quality. The review found that the evidence for surgical abortion procedures provided by MLHPs was lacking. Further, evidence from cohort studies suggested that there was an increase in the risk of failure or incomplete abortion for surgical abortion procedures when provided by MLHPs. However, no statistically significant differences in complications alone, immediate complications or delayed complications were reported when surgical abortion was provided by MLHPs. Concerning medical abortion procedures, the review results suggested MLHPs could safely and effectively carry out these procedures. No significant differences were reported for abortion failure or incomplete abortion. None of the studies included in the SR examined other outcomes of interest such as mortality, quality of care, and access to care. Table 3 presents a summary of findings on various outcomes related to surgical and medical abortion procedures provided by MLHPs compared to doctors.

**Table 3.** Summary of findings for care provided by MLHPs for family planning, contraceptive and other reproductive health care services.

	Outcomes	Relative effect (95% CI)	No of participants	Certainty of the evidence (GRADE)	Plain language summary
Nurses, midwives, doctor assistants, and physician assistants versus doctors					
	**Surgical abortion procedures**				
**RCTs**	Failure/incomplete abortion	RR 2.97 (0.21 to 41.82)	2789 (2 RCTs)	⨁⨁◯◯ Low ^1^	Care provided by MLHPs may increase the chance of the abortion being ineffective or incomplete (more than twice the risk of failure or incomplete abortion for surgical abortion procedures provided by MLHPs when compared to the procedures provided by doctors) (low certainty evidence)
Complications	RR 0.99 (0.17 to 5.7)	2789 (2 RCTs)	⨁⨁◯◯ Low ^1^	Care provided by MLHPs may make little or no difference in complications (low certainty evidence)
Total complications *	RR 3.07 (0.16 to 59.08)	2789 (2 RCTs)	⨁⨁◯◯ Low ^1^	Care provided by MLHPs may increase total complications. However, the wide 95% confidence interval includes the possibility of both increased and reduced risk of total complications (low certainty evidence)
**Observational studies**	Failure/incomplete abortion	RR 2.2 (1.34 to 3.6)	13,715 (3 observational studies)	⨁◯◯◯ Very low ^1, 2^	It is uncertain as to whether care provided by MLHPs reduces the risk of failure of incomplete abortion as the certainty of the evidence has been assessed as very low
Complications	RR 1.38 (0.7 to 2.72)	13,715 (3 observational studies)	⨁◯◯◯ Very low ^1– 3^	It is very uncertain whether care provided by MLHPs reduces complications as the certainty of the evidence has been assessed as very low
Total complications *	RR 1.36 (0.86 to 2.14)	16,173 (4 observational studies)	⨁◯◯◯ Very low ^1– 3^	It is very uncertain about the effect of care provided by MLHPs on the risk of total complications.
	Mortality; quality of care; and access to care	-	-	-	No studies were found that examined these outcomes
	**Medical abortion procedures**				
**RCTs**	Failure/ incomplete abortion	RR 0.81 (0.48 to 1.36)	1892 (2RCTs)	⨁⨁⨁◯ Moderate	Care provided by MLHPs may slightly reduce the risk of failure/ incomplete medical abortion when compared with that provided by doctors (moderate certainty evidence)
**Observational studies**	Failure/incomplete abortion	RR 1.09 (0.63 to 1.88)	1164 (1 study)	⨁◯◯◯ Very low ^1– 3^	It is very uncertain about the effect of care provided by MLHPs on failure/incomplete abortion as the quality/certainty of the evidence has been assessed as very low
	Mortality; quality of care; and access to care	-	-	-	No studies were found that examined these outcomes

*Total complications - incomplete or failed abortion and complications

^1^Downgraded one level due to imprecision and additional one level due to indirectness as studies included were not from the primary healthcare context.

^2^Downgraded two levels due to risk of bias and one level for imprecision (wide confidence intervals)

^3^Downgraded one level due to serious risk of bias

CI, confidence interval; GRADE, Grading of Recommendations, Assessment, Development and Evaluations; RR, risk ratio; RCT, randomised controlled trial; MLHPs, mid-level health providers.

***MLHPs for communicable diseases.*** Two SRs examined the effectiveness of the delivery of antiretroviral therapy (ART) provided by MLHPs in HIV-infected patients ^14, 12^. The reviews included studies mainly conducted in primary health care settings and LMICs. The studies included in the reviews compared ART provided by nurses or clinical officers with doctors. The certainty of the evidence varied for different outcomes, from high to very low quality. However, the evidence for various outcomes was based on relatively few studies. The review reported that there was no significant difference in mortality, with lower rates of losses to follow up at 12 months. Further, no difference in death or number of patients lost to follow up at 12 months was reported when doctors initiated therapy and nurses provided follow-up. The reviews suggested that shifting tasks from doctors to MLHPs may help in potentially reducing costs of ART provision, without compromising on the quality of care and patient outcomes. Table 4 provides a summary of findings reported in the SRs for outcomes related to the initiation and maintenance of ART in HIV-infected patients.

**Table 4.** Summary of findings for care provided by MLHPs for HIV/AIDS and ART.

	Outcomes	Relative effect (95% CI)	No of participants	Certainty of the evidence (GRADE)	Plain language summary
Nurses or clinical officers versus doctors					
**RCTs**	Initiation and maintenance of ART mortality follow-up: 12 months	RR 0.96 (0.82 to 1.12)	2770 (1 RCT)	⨁⨁⨁⨁ High	Initiation and maintenance of ART by a nurse or a clinical officer slightly reduces mortality (high certainty evidence)
	Maintenance of ART death follow-up: 12 months	RR 0.89 (0.59 to 1.32)	4332 (2 RCTs)	⨁⨁⨁◯ Moderate ^1^	Maintenance of ART by a nurse or a clinical officer makes little or no difference in mortality when ART had previously been initiated by a doctor (moderate quality/certainty evidence)
**Observational studies**	Initiation and maintenance of ART death follow-up: 12 months	RR 1.23 (1.14 to 1.33)	39160 (2 observational studies)	⨁⨁◯◯ Low ^2^	Evidence suggests that there may be an increased risk of death when ART is initiated and maintained by a nurse or a clinical officer when compared to a doctor’s care (low certainty evidence)
	Maintenance of ART death follow-up: 12 months	RR 0.19 (0.05 to 0.78)	2772 (1 study)	⨁◯◯◯ Very low ^3^	It is uncertain whether nurse-led care reduced mortality as the quality/certainty of the evidence has been assessed as very low
	Quality of care and access to care	-	-	-	No studies were found that examined these outcomes

^1^ Downgraded by one level for imprecision due to a wide confidence interval

^2^ Rated low because of observational study designs. Not downgraded for risk of bias

^3^ Downgraded by one level for imprecision due to low event numbers CI, confidence interval; GRADE, Grading of Recommendations, Assessment, Development and Evaluations; RR, risk ratio; RCT, randomised controlled trial; MLHPs, mid-level health providers; HIV, human immunodeficiency virus; AIDS, acquired immunodeficiency syndrome; ART, antiretroviral therapy.

***MLHPs for non-communicable diseases.*** Two reviews compared the effectiveness of care provided by non-physician health workers (NPHWs) for patients with non-communicable diseases in primary and secondary health care settings ^15, 16^. The NPHWs included nurses, pharmacists, allied health professionals, and physician assistants. The care provided by NPHWs was compared to that provided by doctors for various physiological measure outcomes, health-related quality of life, and access to care. The evidence assessed was of moderate to high quality. The findings from the two reviews suggested that care provided by NPHWs with varying but high degrees of autonomy and with support was comparable to that provided by doctors for various relevant outcomes. Care prescription by NPHWs significantly improved outcomes such as systolic blood pressure, glycated haemoglobin and low-density lipoprotein levels. Also, the care provided by NPHWs improved health-related quality of life (physical component). However, the mental health-related quality of life was reduced with the care provided by NPHWs compared to that provided by doctors. There was a lack of conclusive evidence on outcomes related to access to care. Table 5 presents a summary of findings for various relevant outcomes related to chronic diseases.

**Table 5.** Summary of findings for care provided by NPHWs for non-communicable disease management.

	Outcomes	Mean difference (MD) (95% CI)	No of participants	Certainty of the evidence (GRADE)	Plain language summary
Non-medical (non-physician health workers (NPHWs)) prescribing compared to medical (doctors) prescribing for chronic disease management in primary care					
**RCTs**
	Systolic blood pressure (mmHg) at 12 months	MD -5.31 mmHg lower (-6.46 to -4.16 lower)	4229 (12 RCTs)	⨁⨁⨁⨁ High	Chronic disease management by non-medical prescribers probably reduces systolic blood pressure (high certainty evidence)
	Glycated haemoglobin (HbA1c, %) at 12 months	MD -0.62 (-0.85 to -0.38)	775 (6 RCTs)	⨁⨁⨁⨁ High	Chronic disease management by non-medical prescribers reduces the glycated haemoglobin levels (high certainty evidence)
	Low-density lipoprotein (mmol/L) at 12 months	MD -0.21 (-0.29 to -0.14)	1469 (7 RCTs)	⨁⨁⨁◯ Moderate ^1^	Chronic disease management by non-medical prescribers probably reduces low-density lipoprotein levels (moderate certainty evidence)
	Health-related quality of life measured with SF-12/36 – Physical component	MD 1.17 (0.16 to 2.17)	2385 (8 RCTs)	⨁⨁⨁◯ Moderate ^2^	Chronic disease management by non-medical prescribers probably improves the health-related quality of life (moderate certainty evidence)
	Health-related quality of life measured with SF-12/36 – Mental component	MD 0.58 (-0.40 to 1.55)	2246 (6 RCTs)	⨁⨁⨁◯ Moderate ^1, 2^	Chronic disease management by non-medical prescribers probably reduces health-related quality of life (mental component) (moderate certainty evidence)
	Mortality	-	-	-	No studies were found that examined this outcome
Access to care	-	-	-	Several studies reported improved access to healthcare at the community level, although the metric to evaluate access was often not described. Data was not reported, and the evidence was not assessed according to GRADE criteria.

^1^Downgraded one level due to serious inconsistency (considerable heterogeneity was found) ^2^Downgraded one level due to indirectness (prescribing component effect on quality of life difficult to determine) CI, confidence interval; GRADE, Grading of Recommendations, Assessment, Development and Evaluations; MD, mean difference; RCT, randomised controlled trial; NPHW, non-physician health worker.

***MLHPs for mental health.*** One SR compared the effectiveness of delivery of care provided by non-specialist health workers (NSHWs) to that provided by mental health specialists in women with perinatal depression ^17^. The NSHWs included midwives, nurses, and community health workers. The studies included in the review were conducted in primary health settings and LMICs. The review found that the NSHWs could effectively deliver psychological interventions for perinatal depression in low-resource settings, particularly where specialist services are both scarce and expensive. The review did not examine other relevant outcomes such as mortality, quality of care, and access to care. The review lacked proper reporting and hence it was not possible to assess the certainty of evidence by GRADE. The SR included nine RCTs involving a total of 14,555 participants. Table 6 briefly presents a narrative summary of the findings reported in the review.

**Table 6.** Summary of findings for care provided by NSHWs for women with perinatal depression.

Outcomes	Impact	Plain language summary
Non-specialist health workers (NSHWs) (midwives, nurses and community health workers) versus mental health specialists		
Perinatal depression assessed using Edinburgh Postnatal Depression Scale (EPDS), the Center for Epidemiological Studies Depression Scale (CES-D), Beck Depression Inventory (BDI), the General Health Questionnaire (GHQ), Hamilton Depression Rating Scale (HDRS) Follow up: range 6 weeks to 3 years	All nine studies reported statistically significant improvements in perinatal depression in the intervention groups compared with control groups. The estimates were presented differently for different measurement scales and at different followup periods.	Only narrative synthesis was conducted for the systematic review and no pooled estimate was available. The results suggested that NSHWs can feasibly provide mental health services leading to improvement in perinatal depression scores, particularly in low-resource settings where specialist services are both scarce and expensive. Certainty of evidence by GRADE was not assessed for it due to the paucity of information in the published SR.
Mortality; quality of care and access to care	-	No studies were found that examined these outcomes

NSHW, non-specialist health worker; EPDS, Edinburgh Postnatal Depression Scale; CES-D, Center for Epidemiological Studies Depression Scale; BDI, Beck Depression Inventory; GHQ, General Health Questionnaire; HDRS, Hamilton Depression Rating Scale; GRADE, Grading of Recommendations, Assessment, Development and Evaluations; SR, systematic review.

***MLHPs for other packages of care.*** This RES did not identify any SRs that assessed the role of MLHPs in the provision of following health services.

- MLHPs for childhood and adolescent health services
- MLHPs for ophthalmic and ENT conditions
- MLHPs for elderly and palliative health care
- MLHPs for emergency medical services

## Discussion

In this rapid overview of SRs, we examined the evidence on the effectiveness of care provided by MLHPs in LMICs for various healthcare domains of India’s CPHC package ^3^, and contextualised the certainty using GRADE approach ^7^. We found that there is some evidence that MLHP-led (auxiliary nurses and midwives, physician assistants, non-physician health workers, and community health workers) care may be appropriate in patients for management of various outcomes in different healthcare domains of interest such as maternal and child health, neonatal and infant health, and communicable and non-communicable disease management when compared to physician or doctor-led care, but the certainty of the evidence for this was mostly low or moderate (barring a few exceptions). As such, while MLHPs can be considered as an alternative to medical professionals for some domains, the certainty of evidence implies the need for building an evidence base and careful evaluation of programs as they are scaled up in India.

There is, however, no synthesised evidence in the form of SRs for childhood and adolescent health services, ophthalmic and ENT conditions, elderly and palliative health care, or emergency medical services. There is a need for conducting well-designed primary studies on these domains to inform future plans for rolling out of MLHPs for CPHC implementation in India.

While we looked at global evidence, the use of GRADE enabled us to contextualise evidence to India. A robust, transparent and comprehensive search strategy was utilised to identify all relevant SRs; and methodological quality assessment of included SRs was conducted using a standardised checklist. Having a wide scope covering multiple CPHC domains enabled the identification of knowledge gaps that could inform relevant stakeholders at the national and state levels.

As part of the RES process, we presented the interim policy brief to engage with key stakeholders, seeking to ensure that the product was robust, relevant, and useful to the target audience. The stakeholders deliberated on the policy brief and provided feedback on the usefulness, relevance, format, and the use of GRADE. Following deliberations with the stakeholders, several changes were made to the policy brief in terms of the use of standardised definitions, the use of more plain language statements, and contextualising evidence to the Indian setting. The inclusion of SRs provided more high-level insight into synthesised evidence around MLHPs. We did not update the reviews and as such we acknowledge the limitation of evidence from primary studies that may have not been included in the reviews.

The use of the WHO definition for MLHP was in alignment with the MLHP term described in the Ayushman Bharat operational guidelines on CPHC. The search for and comparison of global evidence fit the inclusion criteria that were developed in consultation with the requester. The definition of MLHPs as defined by the WHO and in most countries (which we synthesised evidence from), does not include cadres like Ayurvedic, Homoeopathic, Siddha, and other complementary and alternative medicine practitioners who are part of the MLHP programme in India. As such, this limits generalisation and underlines the need for high-quality studies to be conducted in India to assess the effect these workers have, relative to others, in achieving desired outcomes.

We found several gaps in current research on MLHPs. Evidence from SRs of randomised controlled trials is important, but this approach may not be the most appropriate, as they are unlikely to yield data to inform such a complex intervention. Further, substantial primary research may be required on outcomes related to access to care and quality of care. Future studies may consider addressing the implementation aspects as part of the existing healthcare system as well as the cost-effectiveness in LMICs.

There is limited evidence on strategies and facilitators for the implementation of universal healthcare policies and the provision of equitable health care through MLHPs in India. A study in Chhattisgarh that assessed the clinical competence of non-physician clinicians and physicians in the delivery of primary health-care services found comparable levels of competency ^18^. Another study conducted in Chhattisgarh reported that physicians and nonphysician clinicians performed similarly in terms of patient satisfaction, trust, and perceived quality ^19^. In Assam, a three-year rural health practitioner course was developed and implemented to select, train and deploy Rural Health Practitioners (RMPs, a type of MLHP) in sub-centres, which showed significant improvements in the number and the range of services delivered ^20^.

## Conclusion

In conclusion and based on our findings, utilisation of MLHPs for care provision for certain healthcare domains may be applicable, relevant, and feasible in LMICs, including in India. MLHPs such as nurse practitioners, physician assistants, and community health officers will be required for primary care to fill the gaps in access and quality in health services. However, the roles and subsequent training and regulation of MLHPs might be different for several CPHC packages and there is a need for embedded research and robust evaluations in the future.

## Data availability

### Underlying data

All data underlying the results are available as part of the article and no additional source data are required.

### Extended data

Figshare: Extended data.docx. https://doi.org/10.6084/m9.figshare.12401525.v1 ^5^

This project contains the following extended data:

- Appendix 1: Search strategies (since database inception up until March 2019)
- Appendix 2: Updated search strategies (April 2019 to April 2020)
- Appendix 3: Key characteristics of the included SRs
- Appendix 4: Critical appraisal results of included systematic reviews assessed using the AMSTAR-2 checklist

Data are available under the terms of the Creative Commons Attribution 4.0 International license (CC-BY 4.0).
