# Supplementary material for: Mid-level health providers for primary healthcare: a rapid evidence synthesis
Source: F1000Res. 2021 May 10;9:616. Originally published 2020 Jun 16. [Version 2] doi: 10.12688/f1000research.24279.2 (PMC8237370; doi:10.12688/f1000research.24279.2)
Supplement: Peer review history

# Peer review: Reviewer response for version 2

**DOI:** 10.5256/f1000research.56486.r84982

**Published:** 2021-06-25

**Authors:** Guido Bendezu-Quispe

**Affiliations:** 1 Universidad Privada Norbert Wiener, Lima, Peru

The revisions are appropriate.

Is the work clearly and accurately presented and does it cite the current literature?

Yes

If applicable, is the statistical analysis and its interpretation appropriate?

Partly

Are all the source data underlying the results available to ensure full reproducibility?

Yes

Is the study design appropriate and is the work technically sound?

Partly

Are the conclusions drawn adequately supported by the results?

Partly

Are sufficient details of methods and analysis provided to allow replication by others?

Partly

Reviewer Expertise:

Public health, epidemiology

I confirm that I have read this submission and believe that I have an appropriate level of expertise to confirm that it is of an acceptable scientific standard.

# Peer review: Reviewer response for version 1

**DOI:** 10.5256/f1000research.26786.r81670

**Published:** 2021-03-31

**Authors:** Guido Bendezu-Quispe

**Affiliations:** 1 Universidad Privada Norbert Wiener, Lima, Peru

Abstract (Introduction: Provide only necessary information to inform the context of the study. 

 Methods: There is insufficient information about the study methods (quality of the included studies, stakeholder engagement, others). Results: no results are presented on the evaluation of the quality of the evidence. Conclusion: The conclusion does not emphasize that the evidence is of low or moderate quality on the usefulness of MLHPs in primary healthcare, and this should be mentioned). 

 Introduction: 

 The need for a rapid evidence synthesis for the study topic is not explicitly justified. I believe that the word "gratis" in the last paragraph is not suitable. The introduction is focused on India. This topic is suitable for LMIC. Hence, I recommend focusing the narrative of the manuscript on LMIC.    

 Methods:

 Specify stakeholder participation. The methodology describes that a single reviewer performed the screening process (specify the reason for this).

 Results:

 Specify characteristics of the documents included in the analysis. There are tables that could be merged (table 2 to 6) to reduce the number of appendices since the last ones include data that should be presented in the manuscript body.

 Discussion:

 A very brief discussion of the results obtained on the usefulness of MLHPs in primary care was carried out. Authors should discuss and compare the results of the SRs evaluated with the available literature in the study topic. 

 In the document, at times the discussion of the results is oriented to a context of low- and middle-income countries and at other times to the context of India. It would be helpful for authors to target their paper to LMIC with an emphasis on India at the discretion of the authors.

Is the work clearly and accurately presented and does it cite the current literature?

Yes

If applicable, is the statistical analysis and its interpretation appropriate?

Partly

Are all the source data underlying the results available to ensure full reproducibility?

Yes

Is the study design appropriate and is the work technically sound?

Partly

Are the conclusions drawn adequately supported by the results?

Partly

Are sufficient details of methods and analysis provided to allow replication by others?

Partly

Reviewer Expertise:

Public health, epidemiology

I confirm that I have read this submission and believe that I have an appropriate level of expertise to confirm that it is of an acceptable scientific standard, however I have significant reservations, as outlined above.

# Response

**Published:** 2021-04-14

**Authors:** Sandeep Moola

**Affiliations:** George Institute for Global Health, India

Dear Guido, 

 Thank you for taking out time to review the paper and for your valuable comments. We will revise and upload a new version of the article based on your feedback. Thanks again.

 Regards

 Sandeep

# Response

**Published:** 2021-04-26

**Authors:** Sandeep Moola

**Affiliations:** George Institute for Global Health, India

Abstract (Introduction: Provide only necessary information to inform the context of the study.

 Methods: There is insufficient information about the study methods (quality of the included studies, stakeholder engagement, others). Results: no results are presented on the evaluation of the quality of the evidence. Conclusion: The conclusion does not emphasize that the evidence is of low or moderate quality on the usefulness of MLHPs in primary healthcare, and this should be mentioned).

- Thank you for your comments. We have revised the abstract incorporating the necessary changes (page 2).

Introduction:

 The need for a rapid evidence synthesis for the study topic is not explicitly justified. I believe that the word "gratis" in the last paragraph is not suitable. The introduction is focused on India. This topic is suitable for LMIC. Hence, I recommend focusing the narrative of the manuscript on LMIC. 

- We have now provided a statement to justify the conduct of a rapid evidence synthesis on the study topic. We have deleted the word “gratis” in the last paragraph. (page 3) We included some information relevant to LMICs to reflect the context and the nature of the findings of this review.

Methods:

 Specify stakeholder participation. The methodology describes that a single reviewer performed the screening process (specify the reason for this).

- We included relevant information on stakeholder participation (page 4). Thank you for an excellent point on single reviewer screening. We used a single reviewer approach for study screening and selection based on the requirements of the review and resources available at the time. At the time, we considered this as a reasonable approach, as it involved using a single experienced reviewer (SM) for screening with a random verification of a subset of screening records by another experienced reviewer (SB).

Results:

 Specify characteristics of the documents included in the analysis. There are tables that could be merged (table 2 to 6) to reduce the number of appendices since the last ones include data that should be presented in the manuscript body.

- Thank you. We briefly described the key characteristics of the documents under each domain of interest. We note your point about merging tables, but they have been kept separate for each domain of interest to avoid long tables and avoid confusion. We included tables related to essential characteristics of the SRs and the AMSTAR-2 checklist in the extended data file to limit the number of tables in the manuscript body. However, we did try to merge the tables, but the format appeared inappropriate.

Discussion:

 A very brief discussion of the results obtained on the usefulness of MLHPs in primary care was carried out. Authors should discuss and compare the results of the SRs evaluated with the available literature in the study topic.

 In the document, at times the discussion of the results is oriented to a context of low- and middle-income countries and at other times to the context of India. It would be helpful for authors to target their paper to LMIC with an emphasis on India at the discretion of the authors.

- Thank you for your valuable comments on the discussion section. We have revised the discussion section in light of the feedback.

# Peer review: Reviewer response for version 1

**DOI:** 10.5256/f1000research.26786.r76144

**Published:** 2021-01-18

**Authors:** Shradha Parsekar

**Affiliations:** 1 Public Health Evidence South Asia, rasanna School of Public Health, Manipal Academy of Higher Education, Manipal, Karnataka, India

Thank you for providing me opportunity to review this piece of work. 

 Considering the lack of qualified doctors in some of the resource limited settings like India, mid-levels health providers can be an alternative. I must appreciate authors for this work and presenting the findings in a comprehensive manner. 

 I have minor comments: 

1. The focus of the rapid overview of systematic review was on LMICs, however some of the SRs included studies conducted in high income countries (e.g., Barnard et al., 2015 ^1^ included 50% studies from HICs, Weeks et al., 2016 ^2^ included 42 of 46 studies conducted in HICs, similarly, Lassi et al., 2013 ^3^ included studies majorly conducted in HICs). Hence, it would be good if the authors make it clear in the inclusion criteria > context- what percent of included studies within SRs should have been conducted in LMICs. Or explicitly state, the evidence from HICs were eligible considering the statement reported in Discussion section, “ *While we looked at global evidence* *….”.* Secondly, in the result section it was reported, *“All SRs, except one* *^11^* *included studies that were mostly conducted in LMICs”. *However, SR by Weeks et al., 2016 also included studies majorly conducted in HICs.
2. In the Discussion section it was reported, " *While we looked at global evidence, the use of GRADE enabled us to contextualise evidence to India." *Although GRADE help in certainty of evidence, it is worthwhile to consider the contextual factors while contextualising the evidence to India, which I understand was not the focus of this overview of systematic review. 
3. Reference numbering in the extended file and the main text do not match, kindly make necessary edits. E.g., Barnard et al., 2015 is reference number 13 in main text while in supplementary file it is 11.

Is the work clearly and accurately presented and does it cite the current literature?

Yes

If applicable, is the statistical analysis and its interpretation appropriate?

Not applicable

Are all the source data underlying the results available to ensure full reproducibility?

Yes

Is the study design appropriate and is the work technically sound?

Yes

Are the conclusions drawn adequately supported by the results?

Yes

Are sufficient details of methods and analysis provided to allow replication by others?

Yes

Reviewer Expertise:

Epidemiology, Overview of systematic reviews, Qualitative research, Public Health, Systematic review

I confirm that I have read this submission and believe that I have an appropriate level of expertise to confirm that it is of an acceptable scientific standard.

## References

[1] : Doctors or mid-level providers for abortion. *Cochrane Database Syst Rev* .2015; 10.1002/14651858.CD011242.pub2 CD011242 10.1002/14651858.CD011242.pub2 26214844PMC9188302

[2] : Non-medical prescribing versus medical prescribing for acute and chronic disease management in primary and secondary care. *Cochrane Database of Systematic Reviews* .2016; 10.1002/14651858.CD011227.pub2 10.1002/14651858.CD011227.pub2 PMC646427527873322

[3] : Quality of care provided by mid-level health workers: systematic review and meta-analysis. *Bull World Health Organ* .2013;91(11) : 10.2471/BLT.13.118786 824-833I 10.2471/BLT.13.118786 24347706PMC3853954

# Response

**Published:** 2021-03-04

**Authors:** Sandeep Moola

**Affiliations:** George Institute for Global Health, India

Dear Reviewer, 

 Thank you for taking out time to review the paper and for your valuable comments. Sincere apologies for the delay in replying. Will revise and upload a new version of the article based on your feedback.

# Response

**Published:** 2021-04-26

**Authors:** Sandeep Moola

**Affiliations:** George Institute for Global Health, India

The focus of the rapid overview of systematic review was on LMICs, however some of the SRs included studies conducted in high income countries (e.g., Barnard et al., 20151 included 50% studies from HICs, Weeks et al., 20162 included 42 of 46 studies conducted in HICs, similarly, Lassi et al., 20133 included studies majorly conducted in HICs). Hence, it would be good if the authors make it clear in the inclusion criteria > context- what percent of included studies within SRs should have been conducted in LMICs. Or explicitly state, the evidence from HICs were eligible considering the statement reported in Discussion section, “While we looked at global evidence….”.   

- Thank you for raising this point. We have included a statement, which was initially missing, to accurately reflect the nature of the included studies in the review.

Secondly, in the result section it was reported, “All SRs, except one11 included studies that were mostly conducted in LMICs”. However, SR by Weeks et al., 2016 also included studies majorly conducted in HICs. 

- We have deleted the statement.

In the Discussion section it was reported, "While we looked at global evidence, the use of GRADE enabled us to contextualise evidence to India." Although GRADE help in certainty of evidence, it is worthwhile to consider the contextual factors while contextualising the evidence to India, which I understand was not the focus of this overview of systematic review.

 We added relevant information to provide more clarity (page 22)

 Reference numbering in the extended file and the main text do not match, kindly make necessary edits. E.g., Barnard et al., 2015 is reference number 13 in main text while in supplementary file it is 11.

- Thank you for pointing out the error. We checked the main text and the extended data file and fixed the referencing numbering errors that were seen in a couple of places.
